# Freeze-Dried Somatic Cells Direct Embryonic Development after Nuclear Transfer

**DOI:** 10.1371/journal.pone.0002978

**Published:** 2008-08-20

**Authors:** Pasqualino Loi, Kazutsugu Matsukawa, Grazyna Ptak, Michael Clinton, Josef Fulka, Yehudith Nathan, Amir Arav

**Affiliations:** 1 Department of Comparative Biomedical Sciences, Teramo University, Teramo, Italy; 2 Roslin Institute and Royal (Dick) School of Veterinary Studies, Edinburgh, United Kingdom; 3 Institute of Animal Production, Prague, Czech Republic; 4 Institute of Animal Science, Agricultural Research Organization, The Volcani Centre, Bet Dagan, Israel; Fred Hutchinson Cancer Research Center, United States of America

## Abstract

The natural capacity of simple organisms to survive in a dehydrated state has long been exploited by man, with lyophylization the method of choice for the long term storage of bacterial and yeast cells. More recently, attempts have been made to apply this procedure to the long term storage of blood cells. However, despite significant progress, practical application in a clinical setting is still some way off. Conversely, to date there are no reports of attempts to lyophilize nucleated somatic cells for possible downstream applications. Here we demonstrate that lyophilised somatic cells stored for 3 years at room temperature are able to direct embryonic development following injection into enucleated oocytes. These remarkable results demonstrate that alternative systems for the long-term storage of cell lines are now possible, and open unprecedented opportunities in the fields of biomedicine and for conservation strategies.

## Introduction

The possibility of storing blood cells and other cell lines in a dry state though the process of lyophilisation could be of immense benefit for clinical application. However, despite the optimism displayed in some articles [1] and in several major publications describing the preservation of metabolic activity following rehydration of lyophilised erythrocytes [2] and platelets [3], the use of freeze dried blood is still far from clinical application. Furthermore, there are no reports on the lyophilisation of embryonic or somatic cells, spermatozoa being the only nucleated cells that have been successfully stored in a dehydrated state [4].

In addition to the long-term storage of blood cells, it has also been suggested that genetic banks, preferably in the form of cell lines, should be established from animal species threatened by extinction [5]. This proposal stems from the urgent need to stop the biodiversity reduction taking place worldwide [6], and has been inspired by the successful production of the first cloned mammal though nuclear transfer of a somatic cell [7]. However, while somatic cell cloning undoubtedly holds considerable promise for the multiplication of rare genotypes [8], its application is currently limited by the low efficiency in terms of offspring production [9] . Beside this major limitation, very little is known about the majority of endangered species from a reproductive point of view, therefore, it would seem pragmatic to initiate storage of somatic cells with an eye to future improvements in nuclear transfer efficiency. The maintenance of cells in liquid nitrogen is the golden standard for storing frozen cell. Containers with long hold period are largely available nowadays, allowing low cost maintenance of frozen stocks. However, cheaper solutions for long term storage would be welcome.

Recently, our group has demonstrated that somatic sheep cells rendered non-viable by heat treatment retain the potential to generate normal offspring following nuclear transplantation [10]. While the main objective of that work was to demonstrate that radical approaches can be used for nuclear reprogramming of somatic cells, we also established that cell viability is not equivalent to nuclear viability, as previously demonstrated for freeze-dried sperm [4] . As a consequence of these findings, we investigated the capacity of freeze dried cells to maintain nuclear viability, using nuclear transfer as a biological assay.

## Results and Discussion

**Figure 1:**
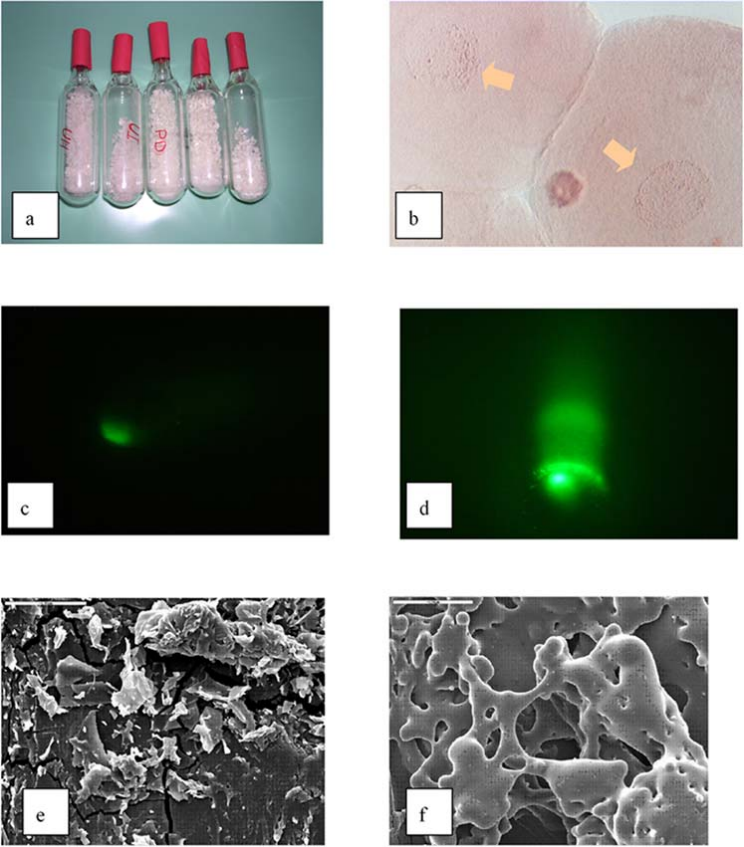
a, ampules with freeze-dried sheep granulosa cells. b, irregular pronuclei with fragmented DNA (10 hours after activation), aceto-orcein staining. ×400. c, d, comet analysis in pronuclear stage embryo reconstructed with a control (fresh) granulosa cell (c), and with a freeze–dried cell (d). ×40. e, f, SEM appearance of the lyophilized cells without (e) and with (f) trehalose. ×350

Sheep granulosa cells lyophilized according to the protocol described for mouse spermatozoa were used as nuclei donor in the first experiments [4]. Following rehydration, the cells, which were non viable and displayed severe membrane damage, were immediately injected into previously enucleated oocytes (n. 354). While a single pronucleus, indicative of normal activation, was found in 90% of sub-samples of oocytes (n. 64) fixed 7–9 hours after injection, none of the cultured embryos entered the first mitosis (data not shown). Cytological and Comet essay revealed extensive DNA damage in all the embryos scored (Figure 1 b, c, d).

These results were not surprising. Spermatozoa, the only eukaryotic, nucleated cells successfully freeze dried to date, are structurally compatible with lyophilisation: they are small cells with a low degree of hydration, and their transcriptionally inactive DNA is tightly packed with protamines [11]. In contrast, the larger size, higher water content, and most importantly, the chromatin organization of somatic cells, are features that render these cells extremely vulnerable to the combined osmotic/dehydration stress imposed by the freeze-drying process.

Although a loss of cellular viability was expected, the frequency and extent of DNA damage was massive and could even be observed by cytological examination (Figure 1b). The enzymatic activity of DNAses is largely influenced by divalent cations [12], and it is possible that the destruction of the cell membrane and sub-cellular compartments associated with lyophilization altered the intracellular ionic homeostasis, leading to the activation of endogenous DNAses. Further chromosomal damage might also have been induced by the exclusion of water from the DNA [13].

These results prompted us to seek means of preventing, or reducing the degree of DNA damage occurring during freeze drying.

The production of threalose by a number of plants, yeast, spores and a range of unicellular organisms confers them the ability to survive in almost total absence of water [14]. The mechanism by which trehalose confers desiccation tolerance is unclear, although trehalose is thought to replace the shell of water from the surface of macromolecules. This action has been demonstrated in proteins [15], while the protective effect exerted by threalose on lipids is apparently due to the inhibition of phase transition temperature of membranes [16]. It has also been shown that diffusion of trehalose into fibroblasts via temporary pores improves survival following deep freezing [17], and remarkably, expression of trehalose in fibroblasts conferred them desiccation tolerance for up to 5 days [18].

Recently, using our device for extra cellular ice crystal propagation, we demonstrated that even extra-cellular threalose protects the cells during dehydration [19]. Consequently, directional freezing to control ice crystal morphology in combination with a Hepes Talp medium containing 0.1 M trehalose, 50% FCS and EGTA was used throughout the remaining experiments for freeze drying sheep lymphocytes and granulosa cells.

The effect of trehalose inclusion in the freezing medium was immediately evident from the morphological appearance of the dry cells observed with Scanning Electron Microscope (SEM). The medium dried in the absence of this sugar showed an irregular splintered-like shape, while the presence of trehalose conferred a smooth and glassy appearance to the dry powder (Figure 1, e–f). Aliquots of freeze dried cells were rehydrated after storage at room temperature for 24 hours and for 30 days, and assessed for membrane and genomic integrity.

**Figure 2:**
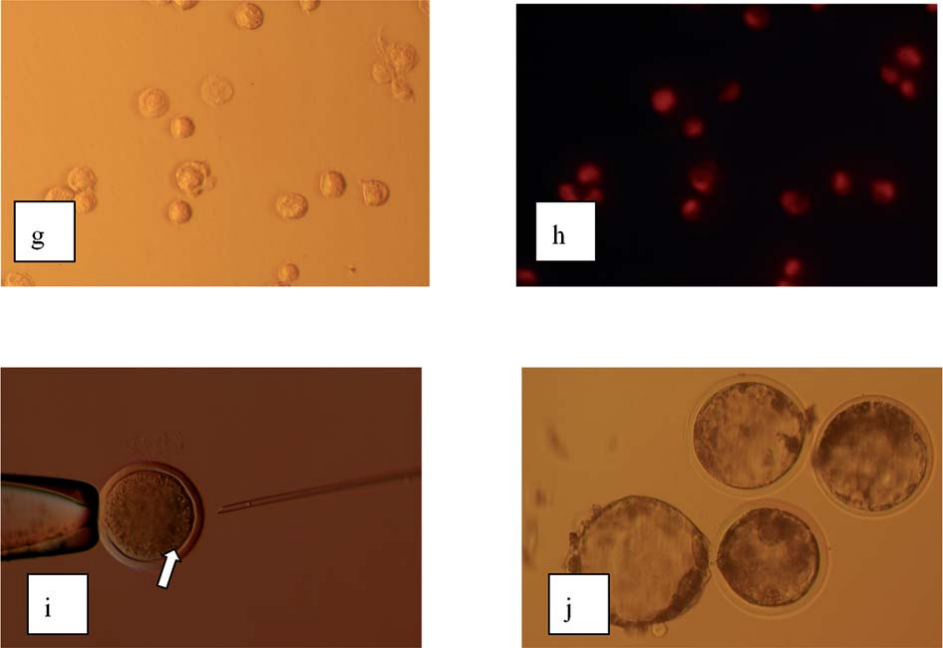
g–h, re-hydrated granulosa cells Propidium Iodine staining (g, bright field; h, epifluorescence) ×150. i, nuclear transfer of freeze-dried granulosa cell×60. j, blastocyst produced from nuclear transfer of freeze dried granulosa cells×40.

Staining with propidium iodine revealed that membrane damage occurred in 100% of the rehydrated lymphocytes and granulosa cells (Figure 2, g, h). Comet essays performed to assess genome integrity showed that trehalose preserved nuclear integrity in 50% of rehydrated lymphocytes and 40% of rehydrated granulosa cells (data not shown). The minor differences observed between these cell may be attributed to the smaller size of the lymphocytes. Despite the higher tolerance of lymphocytes to freeze drying, granulosa cells were used as nuclear donors for nuclear transfer experiments as previous results [10] have indicated an improved development of embryos reconstructed with this cell type.

Freeze dried granulosa cells were prepared and dispatched from the Institute of Animal Science at the Volcani Centre in Israel to Teramo University in Italy by surface mail, and stored in the dark at room temperature for three years before use for nuclear transfer (Figure 1a).

For nuclear transfer, cells were hydrated with Milli-Q grade water, washed twice with 199 medium plus antibiotics and BSA, and immediately injected into enucleated oocytes (Figure 2 i).

Enucleated oocytes injected with freeze dried granulosa cells initiated cleavage at similar rates to control embryos generated using fresh granulosa cells (Table 1). Development to the blastocyst stage (Figure l,j) was lower for freeze dried reconstructed embryos than for control embryos (16% and 21% respectively, P = 0.56; Table 2), most likely as a result of damaged DNA in freeze dried cells. Also, comparing to the controls, the quality of the freeze-dried cloned blastocysts was poor (Table 2). Indeed, comet essay carried out in pronuclear stage embryos at late S phase confirmed that DNA fragmentation occurred in 20% of freeze dried/reconstructed embryos, while was absent in control embryos (data not shown). In this context, it is surprising that 16% of the reconstructed embryos develop to the blastocyst stage when 60% of donor nuclei have obvious DNA damage and raises the possibility that damaged DNA in the donor nucleus might be repaired by factors present in the oocyte. In keeping with this possibility, it has been established that exonuclease and recombination activity both increase during oogenesis, and that fully mature eggs are capable of catalyzing homologous recombination, ligation and illegitimate recombination of exogenous DNA [20].

**Table 1:** In vitro development of enucleated oocytes injected with freeze-dried and fresh, control granulosa cells.

Source of cells	Cultured	2-8 cells	Morula	Blastocyst
Granulosa control	129	43 (33.3%)^a^	31 (24.0%) b	27 (20.9%) c
Granulosa freeze-dried	160	52 (32.5%) a	28 (17.5%) b	25 (15.6%) c

The culture were maintained for 7–8 day in medium SOF plus amino-acids and BSA, with FCS added a day 4. reconstructed embryos were checked every 24 hours for development.

Test χ^2^

a P = 0.88;

b P = 0.17;

c P = 0.56

**Table 2:** Total cell number and ICM and trophectoderm cell counting in control, and freeze dried cells-derived blastocysts.

Total cells	ICM cells	Trophectoderm cells
Control NT blastocysts	28.4±7 a	50.2±12.0 a
78.6±14,8 a		
Freeze dried NT blastocyst	18.5±6 a	39.7±9.0 a
54.29±6.3^b^		

a-b differ significantly for p<0.05

The embryos developed to blastocyst stage were effectively derived from the freeze dried cells and no from parthenogenetic activation of the oocytes. First, all oocytes were effectively enucleated, as assessed by Hoechst staining and UV examination. Second, microsatellite DNA analysis of the cloned blastocysts matched perfectly with the lyophilized donor cells.

### Conclusions

We have demonstrated for the first time that lyophilized cells maintain the development potential when injected into enucleated oocytes. It is also worth noting that the cells used in this study were maintained in a dehydrated state at room temperature for three years, whereas lyophilised spermatozoa were stored for 4 months before Intra Cytoplasmatic Sperm Injection -ICSI [4]. We believe that these findings constitute a major contribution for the long term storage of somatic cells from animals threatened from extinction. However, current efficiencies of SCNT are very low, and despite considerable efforts [21], the controlled reprogramming of a somatic nucleus to re-establish a full totipotency leading to the production of a normal offspring, is still some way off. In short, while this approach will undoubtedly benefit from our findings, re-expanding species by SCNT remains a long-term aim. However, it has been found that defective cloned mammalian embryos, lacking the competence to develop into an entire mouse, can still produce useful stem cell lines [22], [23].

These findings suggest that our results may have important implications for biomedicine in the short term. For example, haematopoietic precursor cells are now widely used as a source of multipotent adult somatic cells for the treatment of degenerative disorders [24]. Cord blood contains a significant proportion of these cells, therefore, it has been suggested that mononucleated cells from this source are stored for future use [25]. Cord blood cells offer advantages over bone marrow cells from related adult donors as they are a stage of development somewhere between embryonic/foetal and adult stem cells, and have a much lower mutation rate than the alternative bone marrow cells [26]. Currently cord blood banking facilities are available in a number of clinical centres, although the use of such facilities is limited due to the associated costs [27]. However, based on our findings, it may be possible to store cord blood cells in a dehydrated state, and when required, these could be re-hydrated and injected into enucleated oocytes for the derivation embryos to be used for the derivation of multipotent cell lines to be used for cell or tissue replacement [28]. However, a great progress is being made on the direct induction of pluripotency to somatic cells through the expression of totipotency-associated genes [29]. Therefore, it is likely that therapeutic cloning, which would benefit from our results, might be obsolete once safe and effective protocols for direct reprogramming of somatic cells will be available [30], [31].

In conclusion, we have demonstrated for the first time that somatic cells stored in a dehydrated state have the capacity to direct embryonic development of enucleated oocytes up to the blastocyst stage. These results represent a substantial breakthrough for the storage of a broad range of cell lines for biodiversity conservation efforts and possibly for biomedical applications.

## Materials and Methods

All chemical were purchased by Sigma, unless otherwise stated.

### Cell collection and freeze-drying

Peripheral blood lymphocytes were isolated from Assaf breed ewes through a Ficoll-Paque density gradient; the purity of the cells (∼80%) was assessed by an automatic cell counter (Pentra 60, ABX, France). Cumulus Oocyte Complexes (COCs) collected from the ovaries of slaughtered Sarda and Assaf ewes were matured for 24 hours in medium TCM 199 plus 10 FCS, FSH, LH and estradiol in incubator at 38,5°C with 5% CO_2_. Expanded COCs were briefly incubated in hyaluronidase (300 USP units/ml, Sigma) and mechanically dissociated into a single cell population. In the first experiments, granulosa cells from Sarda breed ewes were freeze dried according the protocol described for sperm cells [4]. Briefly, 100 μl aliquots of granulosa cells suspended in DMEM supplemented with 10% FCS (1×10^6^cells/ml), were transferred in a glass ampules and snap frozen in liquid nitrogen. Then the ampules were placed on a pre cooled (−50°C) freeze-drier (Freezone Plus 6, Labconco, USA) and lyophilized for 24 hours. Then the ampules were connected to a vacuum pump and flame sealed. In the following experiments kept at the Cryobiology Unit at the Volcani Center the freezing solution was 50% FCS and 0.1M trehalose in Hepes Talp buffer. Two ml samples were frozen using the MTG freezing apparatus (IMT, Israel) at a cooling rate of 5.1°C/min. Cell concentration ranged from 1-6×10^6^cells/ml. After freezing, the samples were stored in liquid nitrogen until inserted to the lyophilizer (Freezone Plus 6, Labconco, USA). Samples were lyophilized for 72 hours, after which, each ampoule was frame sealed and placed into cardboard and stored at room temperature (23–25°C) until use.

### Rehydration

Immediately before use for nuclear transfer, the ampoules were opened and 100 μl, or 2 mls of milliQ water added. After rehydration, cells were washed twice with medium 199 plus antibiotics and BSA before use for nuclear transfer. For each ampoule, viability was assessed on small aliquots of cells by propidium iodine staining; also the extend of DNA fragmentation was assessed with Comet essay in every replicate.

### DNA Integrity

DNA integrity was evaluated using the single cell gel electrophoresis assay (aka comet assay, R&D Systems, USA) according to the manufacturer's instructions. Cells were diluted to a concentration of 105cells/ml in PBS. The cells were combined with molten LM agarose at a ratio of: 1∶10 (v/v), then 75 μl were placed on comet slides. The slides were put in the dark at 4°C for 10 minutes. Slides were then immersed in a pre cooled (4°C) lysis solution for 1 hour at 4°C. Afterward, the slides were immersed in a freshly prepared alkali solution, which consisted of 0.6g NaOH pellets, 250 μl of 250mM EDTA, pH 10.0 and 49.75ml deionized water, for 60 minutes in the dark at room temperature. Finally, the slides were washed in a TBE buffer (Tris base, Boric acid and EDTA) for 5 minutes and then the slides were submerged in TBE buffer in a horizontal electrophoresis apparatus. 1 volt per centimetre was applied for 10 minutes. In addition, each experiment was conducted with two controls; the first one was of cells that were previously treated with 100 μM of hydrogen peroxide for 10 minutes at 2°C–8°C as described in the manufacturer kit (this served as a positive control for the assay showing damaged DNA) and the second control was of fresh cells, indicating endogenous levels of damage within the cells. After the samples were dried they were stained with SYBR green. Scoring was done using a fluorescent microscope (Zeiss, Germany) connect to a digital camera (Sony, Japan) and analyzed using the Image J free software (NIH, USA).

Reconstructed embryos at pronuclear stage (12 hours after activation) were treated in the same way for the detection of DNA damage.

### SEM

For evaluation of the morphology of dry samples Scanning Electron Microscopy was used. The samples were gold plated before being placed in the SEM. The voltage of the electron scatter was 25kv. We captured a 3D picture on a CRT screen.

### Oocyte maturation

Oocytes were matured in vitro in bicarbonate-buffered TCM-199 (Gibco) (275mOsm) containing 2 mM glutamine, 100 μM cysteamine, 0.3 mM sodium pyruvate, 10% fetal bovine serum (FBS) (Gibco), 5 μg/ml FSH (Ovagen), 5 μg/ml LH, 1 μg/ml estradiol in a humidified atmosphere of 5% CO_2_ in air at 39°C for 24 h [32].

### Oocyte enucleation and nuclear transfer

Oocytes were incubated in Hepes buffered 199 medium containing 4 mg/ml of BSA and 7.5 μg/ml of Cytochalasin B and 5 μg/ml of Hoechst 33342 for 15 minutes in incubator. Enucleation was carried out in Hepes buffered 199 medium plus BSA and Cytochalasin B with a Narishighe micromanipulator. Enucleate oocytes were allowed to recover from Cytochalasin B treatment then directly injected with freeze-dried cell. Reconstructed oocytes were activated in Hepes buffered medium 199 containing 5 μg/ml Ionomycin for 5 minutes, then incubated in SOF medium plus antibiotics and BSA containing 10 mM Dimethylaminopurine and 7.5 μg/ml Cytochalasin B for 3–5 hours.

### Embryo culture

Reconstructed embryos were transferred into 20-μl drops of SOF enriched with 1% (v:v) basal medium Eagle (BME)-essential amino acids, 1% (v∶v) minimum essential medium (MEM)-nonessential amino acids (Gibco), 1 mM glutamine, and 8 mg/ml fatty acid-free BSA (SOFaaBSA). Zygote cultures were maintained in an humidified atmosphere of 5% CO_2_, 7% O_2_, 88% N_2_ at 39°C, and the medium renewed on Day 3 and Day 5 of culture [32]. Embryonic development was monitored every 24 hours, and arrested embryos were fixed (acetic acid/methanol 3∶1) for 24 hours, then stained with 2% aceto-orcein. Aliquots of reconstructed embryos were fixed (13–16 hours post activation) and processed for Comet analysis.

### Differential staining of blastocysts

Cloned blastocysts from both groups were incubated in 500 μl of solution 1 (PBS with 1% Triton X-100 and 100 μg/ml propidium iodide) for 20 s. Blastocyst were then directly transferred to 500 μl of solution 2 (100% ethanol with 25 μg/ml bisbenzimide Hoechst 33258) and stored at 4°C overnight. Blastocysts were then mounted onto a microscope slide in a drop of glycerol and flattened with a cover slide. Cell counting was performed directly on an inverted microscope fitted with an ultraviolet lamp and excitation filter (460 nm for blue and red fluorescence).

### DNA microsatellite analysis

Cloned blastocysts were transferred in 200 μl of SOF medium and snap frozen in liquid nitrogen. DNA was extracted from the cloned blastocysts and from the donor cells using the kit Genomix, according with the manufacturer instruction. Genomic DNA from cloned embryos and donor cells have been amplified through a multiplex PCR for the following satellites: OarCP49, FCB11, OarAE129, FCB304, INRA063, MAF214, CSRD247 e HSC. These loci are part of a standard panel recognized by the International Society for Animal Genetics (ISAG).

### Ethical statement

All the experiments wee carried out in vitro, using ovaries collected from the slaughterhouse, without any experimental animals, therefore, we did not need to receive ethical approval from the local University Ethical Committee.
